# Dislocation of an Eye-Ball

**Published:** 1876-01

**Authors:** A. W. Calhoun

**Affiliations:** Professor Diseases of the Eye and Ear in Atlanta Medical College


### DISLOCATION OF AN EYE-BALL, WITH REPLACEMENT AND COMPLETE RECOVERY OF VISION.

By A. W. CALHOUN, M.D., Professor Diseases of the Eye and Ear in Atlanta Medical College.

The case is not that of a human being, but of a dog. But I report it as of interest in itself, and as being an exact representation of the injury as occurring in the human subject.

During the later part of the summer, Dr. J. M. Johnson was called to the house of a certain judge, the messenger telling him that a favorite dog, belonging to the children, had lost an eye, and needed immediate attention. I was requested to accompany the doctor, and upon arriving at the residence, we found the history of the injury and the condition of the little animal about as follows: The dog, a small Esquimaux, was playing with a large New Foundland, when the latter took it upon himself to seize the diminutive head of his companion between his jaws, and pressed with such vigor as to force the left eye ball from its socket.

We found the little animal apparently in the greatest misery, the ball resting upon the cheek, and the lids contracted so tightly behind it as to enable us to see the whole of the posterior portion, and thus also preventing completely the voluntary return of the ball to its normal position. The optic nerve was put upon the stretch to its very utmost, producing total blindness, so far as we could judge. The cornea, being exposed for several hours to external influences, had assumed an opaque appearance, being in itself a cause of blindness.

Holding the dog firmly, and grasping the protruded ball between the thumb and the first two fingers of the right hand, and with the fingers of the left separating the contracted lids as much as possible, the ball was slowly and gently compressed and directed backwards, when, after a few moments of such pressure, the globe glided into its place, reminding one very much of the noise produced at the moment a dislocated limb springs back into its articulation, with its peculiar characteristic snapping noise. Our little patient gave immediate evidence of the greatest relief, and bounced across the room to the delight of all present, giving unmistakable signs of the already partial restoration of vision in the injured eye. Upon inquiry, a few days later, we were told there were no symptoms of anything unusual having ever existed with the eye, the vision and position of the ball being as perfect as that of the other.

In former days, when the old-fashioned mode of fighting (gouging) was so much in vogue, the same thing oftentimes followed, as did in the case of the dog. That combatant was considered the most successful, who could first press one or more fingers behind one of his opponents eye balls, and force it from its bed, and leave it exposed upon the cheek, an evidence of victory to the surrounding and cheering crowd. Strange that so little serious and permanent injury resulted from such treatment of the eye. But the optic nerve is susceptible of being drawn out to a very considerable extent, without rupture, and can even remain in this *stretched* condition for some time, suffering only a partial benumbing of its fibers, which passes off in due time upon removing the strain. Why the nerve should have such power is readily seen, when we consider the wide range the eyeball has, carrying the optic nerve with it in every movement. Behind and around the ball is a quantity of fine, loose areolar tissue, which not unfrequently takes on an inflammation, swells enormously, and thereby forces the ball very far in advance of its natural position. Nature has made provision for the optic nerve under such circumstances, in giving it the great elasticity, and the power of rapidly recovering from such apparently violent injury, as might be supposed to take place under the above condition of things.

'Tis well known that the muscles around and governing the movements of the ball can be drawn out to an amazing degree, and again assume their normal length and function. They rarely become ruptured, and always afford protection to, and, to some degree, prevent the same injury taking place in the nerve.

Some very interesting reports have been given of dislocated balls, produced in various ways.

I recollect seeing a few years ago, in a foreign hospital, a very old, decrepid, Polish Jew brought in with his left eye-ball thoroughly dislocated, and the only history that he could give of the accident was, that while turning himself in bed the night previous, the ball "suddenly jumped out." The lids must have been completely relaxed, so as to afford no resistance to the ball as it was pressed forward by some of the surrounding muscles contracting at the moment of turning. The ball was easily replaced, and patient dismissed.

Von Langenbeck, of Berlin, reports a case (A. F. O. xiii., ii., 447) of fracture of the superior maxillary, where the right eye-ball was displaced from its socket and lodged in the antrum of highmore. The replacement was easy, but subsequently became atrophied as the result of a plastic operation to supply some of the parts destroyed at the time of the accident.

Von Becker, of Heidelberg, also reports a case (A. F. O. xii., ii., 289) in which a blow from the horn of a cow, dislocated the eye so far back into the orbit that, in seeing the case sometime afterwards, he at first supposed an enucleation of the ball had taken place. The conjunctiva overlapped the eye so completely as to hide it from view, but in pulling this membrane aside, the ball could be seen, and the patient, by thus exposing it, had tolerable vision. As the time that had elapsed since the injury was considerable, allowing extensive adhesions, it was not thought prudent to attempt to bring the ball into its natural position.

A dislocation of an eye-ball, then, by no means is equivalent to the destruction of the same; but by immediate and proper attention, it can be replaced with the retention of every function intact. Of course the ball must be handled carefully, else the pressure exercised in its replacement will result in some accident to the interior of the eye.

